# Non Coding RNAs and Viruses in the Framework of the Phylogeny of the Genes, Epigenesis and Heredity

**DOI:** 10.3390/ijms13010477

**Published:** 2012-01-04

**Authors:** Daniel Frías-Lasserre

**Affiliations:** Institute of Entomology, Metropolitan University of Educational Sciences, Avenue J.P. Alessandri 774 Ñuñoa, Código Postal 7760197, Santiago, Chile; E-Mail: daniel.frias@umce.cl; Tel.: +56-2-2412457; Fax: +56-2-2412699

**Keywords:** non-coding RNAs, epigenesis, imprinting, RNA viruses, coevolution

## Abstract

The origin of genes is one of the most enigmatic events in the origin of life. It has been suggested that noncoding (nc) RNA was probably a precursor in the formation of the first polypeptide, and also at the origin of the first manifestation of life and genes. ncRNAs are also becoming central for understanding gene expression and silencing. Indeed, before the discovery of ncRNAs, proteins were viewed as the major molecules in the regulation of gene expression and gene silencing; however, recent findings suggest that ncRNA also plays an important role in gene expression. Reverse transcription of RNA viruses and their integration into the genome of eukaryotes and also their relationship with the ncRNA suggest that their origin is basal in genome evolution, and also probably constitute the first mechanism of gene regulation. I am to review the different roles of ncRNAs in the framework of gene evolution, as well as the importance of ncRNAs and viruses in the epigenesis and in the non-Mendelian model of heredity and evolution.

## 1. Introduction

The origin of genes, the genetic code and the genetic program, are puzzling events in the origin of life. At present, we know that in the genome of eukaryotic organisms there are different regions. Scattered throughout the genome are the regions of DNA coding for proteins, which in humans corresponds to approximately 2% of the genome. In the remaining 98%, the retroviral genes and also broad segments of DNA that code for non-coding (nc) RNAs are located.

Once it was established by Avery *et al.* in 1944 that the DNA corresponded to the hereditary material, the focus was mainly on the DNA as the central molecule in the hereditary models. This concept was consolidated as the Central Dogma of Molecular Biology [1]. Thus, a deterministic and reductionist inherited pattern emerged that not only had influence on molecular biology, but also on the population genetics and organic evolution. Under this scenario, both the genetic code and the genetics program were mainly associated with the coding DNAs.

At the dawn of genetics as an experimental science, the Mendelian gene was considered a symbol and a fixed and discrete heritable trait, indivisible by recombination, a concept that changed between the years 1955–1959 with Seymour Benzer’s studies on T4 phages in *Escherichia coli* [2,3]. Benzer found that the gene could recombine internally and, unlike the Mendelian gene, the same segment of DNA could be defined based on different criteria such as mutation, recombination and function.

With the first DNA sequencing effort by Frederick Sanger, it was clearly demonstrated that the gene is a nucleotide sequence that encodes proteins, in which genes encode for the amino acid sequence of the primary structure of proteins [4]. Before the human genome project, the hypothesis was that the human species should have about 100,000 genes. However, surprisingly human genes are about 30,000. About 10,000 more than in *Drosophila melanogaster*, and in rice about 15,000 genes more than human have been described. There are discrepancies between the number of coding genes and the size of the genome, these paradoxical facts are explained because the more variable and abundant regions are located in the non-coding regions. Thus, the proteomes of the higher organisms are relatively stable; humans and mice share 99% of protein coding genes and between human and chimpanzee structural genes show 98% similarity [5,6]. Therefore, interspecific differences have to be located in the non-coding regions. Then, some questions arise: what is a gene? And also where is the regulatory program?

According to a holistic concept, a gene is a useful sequence of nucleotides. Repeated sections of non coding DNA involved in the formation of centromeres, telomeres and replication origins should be called also genes [7–10]. Also, it has been proposed that areas of the DNA coding for ncRNAs should also be called genes [11]. Under this framework, virtually the entire genome is useful to the organism. The genetic program important in the formation of a multicellular organism would be located in the non-coding area, through fine processes of genetic regulation via proteins, retroviruses, transposable genetic elements, introns and ncRNAs. Recently, a model has been proposed based on a hypothetical “master development program” for multi-cellular organisms where the DNA sequences are protected by the heterochromatin and transcribed into “Control Keys” that consist of nuclear messenger RNAs that regulate high-level transcription factor genes [12].

Genes were considered a continuous segment of nucleotides, until 1977 when Roberts and Sharp found that adenovirus transcribed RNA was fractionated into separate segments showing that genes are split into exons and introns. Later, in 1989, Thomas Cech and Sidney Altman discovered the catalytic properties of RNAs. They found that group I introns can be excised in the absence of any proteins acting as an enzyme (ribozyme).

The discovery of ribozymes not only changed the central position of the DNA in the dogma of molecular biology but also the paradigm that DNA was the precursor molecules of life, and spurred the idea of a primitive RNA world [13]. This hypothesis also contributed to the discovery of a huge variety of ncRNA with different functions in the three domains of the tree of life. Many classes of the non-coding RNAs have been discovered in the human genome and constitute about 60% of the total transcriptional output [14–16].

These ncRNAs, together with DNA methylation and acetylation of histones, are epigenetic factors that have changed the understanding of genome structure, the concept of the gene, regulation of gene expression and the mechanisms that account for organic evolution. The questions that arise are: what is the origin of ncRNAs? What is the relationship between ncRNAs with transposable genetic elements and viruses? What impact have they had on the legacy and evolution of species? And, should viruses be considered as a fourth domain? This article aims to answer these questions.

## 2. Results and Discussion

### 2.1. The Discovery and the Origin of Non-Coding RNAs

The first ncRNAs were detected by Robert and Sharp in 1977 as micro (mi) RNAs during the discovering that structural genes were split into exons and introns. At first it was thought that introns have no function. However, since the discovery of messenger RNA splicing in adenoviruses [17], the alternative splicing of introns explain the production of several protein in basis to the same mRNA. Thus, it is estimated that the human genome has about 30,000 genes but about 75,000 different kinds of proteins.

The first ncRNAs with a function were described in 1993 in *Caenorhabditis elegans* when it was found that small miRNAs (constituted of about 22 nucleotides) were important for the appropriate timing of post-embryonic development [18,19]. Actually, it is known that around 98% of all transcriptional output in humans is ncRNAs [6]. Recently, the presence of ribozymes has been revealed in the ncRNAs of mammalian genomes [20–23]. One of these ribozymes, discontinuous hammerhead ribozyme has been initially described in viroids [24] and then also in eukaryotic genomes—plants and animals vertebrates and invertebrates. Some of these ribozymes have also been associated with retrotransposable elements [23]. The similarity showed between some those ribozymes suggests a viroid origin. It has also been reported that many of the ncRNA come from introns. Introns were inserted into preformed genes late in eukaryotic evolution [25].

Another hypothesis suggests that the first introns probably originated about 3500 million years ago in eubacteria and were restricted to tRNAs, and were mobile and self-splicing. From these spliced introns would have evolved spliceosomal from a common ancestor of eucaryotes and archaebacteria about 1700 million years ago with the origin of the nucleus and after the origin of mitochondria [26]. Surprising homology between viruses with very distantly related hosts by phylogenetic analysis suggest that genes might have flowed from viruses to eukaryotic chromosomes [27]. Viral oncogenes contain introns that are important in the expression of these oncogenes by alternative RNA splicing in papillomavirus genome [28], these early primary transcripts are bicistronic or polycistronic, and each contains exons and introns [29].

Similar sequences have been described between reverse transcriptase viruses, transposable elements, and mitochondrial introns [30]. Similarly, on the basis of genetic homology it has been discovered that a virophage represents a probably common origin between a DNA viruses and eukaryotic DNA transposons. The virophage parasitizes a giant virus and encodes 20 predicted proteins, these facts suggest that transposons may have originated from ancient relatives of giant virus, and thereby influenced the evolution of eukaryotic genomes [31]. Also there are DNA virophages that parasitize species of bacteria, archeae and eukarya dependent in their reproduction of giant viruses [32].

The discovery of viroids by Diener [33] led to the modification of the paradigm that considered viruses as the smallest inciting agents of infectious diseases. Viroids, single-stranded circular RNAs of 246–375 nucleotides in length able to infect certain plants, are currently the lowest step of the biological scale. They are located in the nucleus of the host cell and probably interfere with the removal of introns and splicing exons, acting as ribozyme. The genomes of viroids are not translated, and are able to self-cleave through hammerhead ribozymes [33,34]. Viroids have nucleotide sequences similar to introns that are removed. They are parasites of plants and can be transmitted through seeds and parasite vectors. These peculiar features of viroids, along with the presence of ribozymes in some of them have been considered as molecular fossils that originated in a precellular environment of RNA, whose existence was probably before the emergence of life based on DNA and proteins [35].

In mammals, many miRNAs derive from repeat elements such as LINES and Alu [36–38]. Alu elements were originally thought to represent junk having no biological functions, nevertheless it has been postulated that ALU elements in human mRNAs are miRNAs targets, and also that other classes of ncRNAs such as short interfering (si)RNAs derive from other small RNAs [37].

The resemblance of these ribozymes in viruses and also in organism belonging to the three domains of life suggests horizontal gene transfer. For example, the similarity of some vertebrate ribozymes with those widespread within mobile genetic elements in trematodes suggests that recurrent genetic horizontal transfers could have taken place from parasites to hosts [23].

### 2.2. Functions of Non Coding RNAs

In recent years a wealth of information has accumulated about ncRNAs. They are characterized according to their function, location, length and also in relation with nearest structural genes. The non-coding RNAs have roles in a great variety of processes, including transcriptional regulation, chromosome replication, mRNA processing and modification, mRNA stability and translation, sex determination and even protein degradation and viral defense [39–41]. For example, the alteration or loss of non-coding RNAs results in modification in developmental processes and diseases [42].

When their function is known, ncRNAs can also be classified by whether they act in *cis* or *trans. Trans*-acting functions are associated with shorts ncRNAs (18–300 nt), such as siRNAs, micro (mi) RNAs, piwi-interacting(pi)RNAs and short nucleorar (sno)RNAs. Some miRNAs having both oncogenic and tumor-suppressive functions are dysregulated in many types of cancer. miRNAs also interfere with metastasis, apoptosis and invasiveness of cancer cells. [15,40,43,44]. In recent years, miRNAs have been described in mammals playing a role in the regulation of neural plasticity, synaptic plasticity regulation of learning and memory and cognitive capacity by regulating dendrite morphogenesis during early development [45,46]. Endo-siRNAs show a significant increase during an early stage of training and have been implicated in neuropsychiatric diseases [47].

Viruses can also generate viral miRNAs that disturb normal host cell functions. About 20–25% of human cancers have a known viral etiology. Expression of the viral oncogenes are regulated by alternative RNA splicing and several species of mRNAs can be derived from a primary transcript of a single viral oncogene to encode different oncoprotein [28,48,43]. Also, viral ncRNAs have been shown to play an important role in virus-host interplay to facilitate virus replication [49].

In opposition, *cis*- acting functions have so far only been associated with macro or long nc (lnc) RNAs, which can be up to several hundred or thousand nucleotides long, about 200–2800 nt [50,51]. In the eukaryotic genome and, especially in mammals, there are thousands of lncRNAs that are expressed in different cell lines and tissues. These lncRNAs have different regulatory functions, principally X chromosome inactivation by hetrochromatinization (*Xist* gene in *cis*), [52], regulation of transcriptional and post transcriptional pathway programming, regulation of mRNA splicing, epigenetic gene activation in the regulation of *Hox* genes, in genome imprinting and as enhancers of gene expression [42,53–56]. LncRNAs have functions also in telomere-length of chromosomes [57–60].

### 2.3. The Non-Coding RNAs and Their Relationship with Epigenesis and Genomic Imprinting

The concept of epigenesis was born to oppose the theory of preformism. This term was coined in 1942 by Conrad H. Waddigton to explain how an adult can be formed from a zygote, through development, by cell differentiation and gene regulation. The epigenesis is defined as heritable changes in the expression of genes that do not involve a change in DNA sequence but only changes in the chromatin. These changes alter the ability of genes to respond to external signals [50]. Thus epigenetic control of gene expression allows heritable or transgenerational changes in gene expression without the need of mutations, not necessarily following Mendelian patterns of inheritance. Epigenesis can thus, for example, explain the hereditary basis of the classical concepts of norm of reaction and phenotypic plasticity. Classically, these concepts are related to a variation with an exclusively environmental base. However, now we know very well that epigenetic regulation of gene expression is accomplished by DNA cytosine methylation, histone acetylation, chromatin remodeling, and also involves the ncRNAs [61]. In mammals, DNA methylation is involved in normal cellular control of expression, and hypermethylation can lead to silencing of tumor-suppressor genes in carcinogenesis [62].

In recent years, growing evidence has emerged that non-coding RNAs play essential roles in the regulation of gene expression in plants and animals, vertebrates and invertebrates [50,63,64]. MiRNAs can negatively control their target gene expression posttranscriptionally. Recently, the expression of miRNAs has been linked to cancer development, and miRNA profiles can be used to classify human cancers. In mammals, as in *C. elegans*, miRNAs can function to prevent cell division and drive terminal differentiation. An implication of this hypothesis is that down regulation of some miRNAs might play a causal role in the generation or maintenance of tumors [65].

The concept of epigenesis also extends to DNA and RNA editing. RNA editing is an epigenetic regulatory mechanism that was discovered from the unicellular protozoa *Trypanosoma* mitochondria. A number of genes are expressed in an unconventional manner, the nucleotide sequence of primary transcripts is modified post-transcriptionally through the insertion or deletion of Uridine. These nucleotide alteration was coined as RNA editing [66,67]. RNA editing has been detected in unicellular and multicellular eukaryotes but not in prokaryotes. After this discovery, it was thought that this process affects only mRNAs, but now it is known that the editing also occurs in tRNAs, rRNAs and miRnas [68–72]. In humans RNA editing is a change of adenosine to inosine mediated by the enzyme adenosine deaminase, acting on double-stranded RNA, where the inosine acts as guanosine [70,71]. In mammals another kind of RNA editing has also been described consisting in a change of cytosine to uridine [73].

RNA editing expands the possibilities for expression of the epigenome by the production of different proteins from a single structural gene [73]. The RNA editing involves not only post-transcriptional changes but also phenotypic changes and therefore greater phenotypic plasticity of the organism against its environment. Thus, RNA editing generates variation of the epigenome contributing to the adaptation of organisms to their environments. In plants, there are data that show that RNA editing mostly affects evolutionarily conserved RNA codon position. These findings support the hypothesis that natural selection has contributed to selective fixation of certain RNA editing sites [74]. In animals and in particular in mammals, RNA editing is especially active in the brain, altering codons in mRNAs. RNA editing and other functional ncRNAs could be involved in diseases and also in brain development, brain plasticity and brain evolution [75].

The epigenome is also very important for genomic imprinting consisting in an asymmetric expression of genes with different paternal origins. Thus, genes are silenced when inherited via sperm or via egg. The term is used in relation to genes that are maternal inherited but silenced when paternally inherited. Thus, genomic imprinting is an epigenetic mechanism that induces parental specific gene expression in diploid eukaryotic cells [76,77]. The genes belonging to both mother and father can be expressed in a cell. However, due to imprinting, the expression of some genes are restricted to only one of the two parental chromosomes causing sex-specific changes in gene expression or chromosome behavior. The gametic DNA methylation mark is then maintained on the maternal or paternal allele [78]. These allelic marks are transmitted through the generations and do not follow a Mendelian pattern of inheritance.

Genomic imprinting changes gene expression between parents without altering DNA sequence. However, these DNA sequences are crucial to score an imprinting domain that are composed of non-coding DNA sequences. The missing of these sequences implies the lost of genome imprinting [50,64]. NcRNA such as, snoRNAs, miRNAs, piRNAs, and siRNA are very important in genomic imprinting. SiRNA is a highly conserved post-transcriptional silencing mechanism in which double stranded RNAs are processed to form guides for the degradation of complementary RNA transcripts through an RNA silencing complex [79–81]. The production of non-coding RNA has been described at multiple imprinted regions in both mammals and plants [81,82].

Much evidence has accumulated showing that imprinted genes can influence animal behavior. A paradigm in this way in humans are the Angelman and Prader-Willi syndromes that included neuroendocrine problems. Thus, children who inherit a deletion on chromosome 15 in the same locus from their father show a behavior different from these children that inherit these same altered locus from their mother [83]. Also in Turner Syndrome, girls that inherit their single X chromosome from their mother present different social dysfunction that who inherit the X chromosome from their father [84]. In mice it was described that genomic imprinting is related with the maternal care of offspring [85].

### 2.4. The Non-Coding RNAs and Their Relationships with Viruses

RNA viruses have a simple organization. They carry short RNA sequences, which are surrounded only by a protein or lipoprotein capsule and do not have a metabolic system that allows them to reproduce itself autonomously. Indeed they do need the host for full metabolic activity. Viroids and virusoids are even simpler because the RNA is devoid of lipids and proteins and has no information for encoding proteins, and their genomes are similar in a conserved sequence to group I of introns that are found in nuclear rRNA genes, mitochondrial mRNA, rRNA genes, and chloroplast tRNA genes. The hallmark of these elements is a 16 nucleotide phyllogenetically conserved RNA sequence [86].

According to their structure, viruses are more similar to a set of molecules than with an organism, placing them among the living and the nonliving. However, viruses have a hereditary material, an universal genetic code and structural genes similar to bacteria, archaea and eukarya, but they have not been considered in the origin of the first life-forms on our planet, nor as a domain in the tree of life. One reason is the dependence of the metabolic system of a host cell for their survival and reproduction. Additional evidence for not having a “virus domain” includes the idea that viruses are derived from the genome of eukaryotes, and have a very unstable genome [87]. All this lead viruses to be disregarded as important elements from the neo-Darwinian evolutionary theory and also in the endosymbiotic theory of Lyn Margulis. However, recently there are many convincing arguments on the influence of viruses not only in the root of the tree of life but also in all its ramifications of the different domains of bacteria, eukaria and archeae, calling for a need to include viruses in the tree of life [88–90].

Viruses can recombine their genetic material with each other as was classically demonstrated by Benzer [2,3] using mutants of bacteriophage T4. Genic recombination in RNA viruses has been known to occur in the poliovirus since about fifty years ago and has been used as a model to investigate molecular mechanisms of recombination in a single stranded genome of positive polarity [91]. Recombination plays an important role in the evolution of RNA viruses by generating genetic variation, by reducing mutational load and by producing new viruses by recombination between different strains [92].

Genetic recombination is a universal phenomenon in all living systems and is also one of the fundamental conditions of sexuality. In the most simplistic sense, sexuality is the transfer and recombination of genetic material between organisms of a species. From this point of view, sexuality occurs between the genetic material of different strains of viruses and also between viruses and the genetic material of their hosts.

### 2.5. Sexuality Between Viruses and Related Genetic Elements

Classically it has been demonstrated that viruses may be intermediaries in the sexuality of strains of *Salmonella typhimurium* [93]. The material transferred by viruses (transduction) is incorporated into the host bacterium and inherited through generations. It has been shown that transduction of genes may be restricted or generalized. Restricted transduction occurs in occasions when the virus vectors are inserted into specific places of host chromosome and can transduce genes or very specific pieces of DNA adjacent to the insertion site [94]. Such type of transduction is also described in bacteriophage λ and *E. coli* K12 strain. In this case, λ is inserted between the gal and biotin genes, where it is as a prophage, but when it disintegrates the host genome gal of K12 gene it is part of the DNA of the virus and can be transduced to a different strain of bacteria lacking this gene when infected by the virus. In generalized transduction viruses prophages are inserted anywhere along the genome and can transduce any gene of the host. An example of generalized transduction is what happens in lysogenic strains of *Salmonella typhimurium* attacked by phage P22 [94].

Horizontal gene transfer mediated by viruses and bacteria have been detected in eukaryotes. In *Saccharomyces cerevisiae*, recombination between retrotransposons is a source of chromosome rearrangements and a mechanism of genome evolution in this specie [95]. Recent studies show recombination between retroposons and exogenous retroviruses [96]. Extensive sequence similarity in various organisms showed that the capsid protein and RNA-dependent RNA polymerase genes from viruses have widespread homologies transversally in the genomes of eukaryotic organisms. Sequence comparison and phylogenetic analysis suggest that these genes were likely transferred horizontally from viruses to eukaryotic genomes and are also functional in the recipient genomes. Horizontal transfer of double-stranded RNA viral genes is widespread among eukaryotes and may give rise to functionally important new genes, thus entailing that RNA viruses may play significant roles in the evolution of eukaryotes [97].

In particular, the persistent endogenous retroviruses in a long evolutionary time scale on a specific host can explain the origin of numerous basic and highly complex functions of life such as the origin of DNA-based genomes and DNA replication, eukaryotic nucleus, adaptative immune system and interference RNAs and mammalian placenta and viviparous birth [88,98] and also all the different class of ncRNAs.

## 3. Conclusions

The affinity in terms of molecular structure, transmission and recombination of genetic material between viruses, transposons, and introns demonstrates the viral origin of the latter elements and strengthen the hypothesis of an initial world of RNAs. It is known that many retrotransposons and retroviruses differ only in the absence of a capsule. I concord with the idea that RNAs viruses should be seen as the first manifestations of life which culminated in the biochemical evolution in a naked RNA world that preceded the origin of the first cells. With the discovery of the ncRNAs and ribozymes, these were located in the center of the central dogma of molecular biology displacing the DNA. The most plausible hypothesis now in the emergence of the first molecules pioneers that life revolves around an RNA virus not dependent of DNA in their replication. Many of the functions within living cells such as replication, transcription and repair as well as their fine-tuned regulatory order are now known to also be of viral origin [80,99].

If the origin of introns and retrotransposable elements are viral, then ncRNAs, in base to its homology with these elements, have also a viral origin and are part of the genome in the different species of the tree of life. Thus, the ncRNAs, in the form of introns, by splicing of mRNA, regulates the genetic expression. Also, the other forms of small and ncRNAs, coded in the redundant DNA and located between structural genes, have many important roles in the organism.

The molecular evidence demonstrates that the ncRNAs are scattered in the species that constitute the three domains of the tree of life. These ncRNAs are endosymbiont coadapted molecules with the genome of hosts and are the product of molecular coevolution from the origins of the first cells. The importance of RNA and DNA viruses in the evolution of their host as a persistent evolutionary force has been analyzed extensively by Villarreal [88,98], this analysis revealing that not only the vertical Mendelian inheritance has been important in the organic evolution but also the horizontal virus-mediated gene transfers have been a fundamental evolutionary force in the diversification of species and biodiversity. These horizontal gene transfers may even be the cause of disease. In fact, increasing evidence for a relationship between carcinogenesis and infections by helminths has accumulated during the last decades; trematode infection could affect the host genomic stability and promote activation of introns of host genes involved in tumor progression [23].

The natural viral transduction between the species that constitute the tree of life and genetic homologies between distant organisms suggest that the genome of living organisms, particularly multicellular is a “fluid mosaics of genetic information from different sources” [10]. This fact also suggests that all organisms are naturally transgenic.

The discovery of ncRNAs has represented a major shift in the way of conceiving genetic variation in natural populations. Before the advent of the ncRNAs, the norm of reaction and phenotypic plasticity were considered adaptive but it was thought they had no a hereditary basis. But, now we know that these phenomena have a strong epigenetic base and can be inherited, explaining the emergence of morphological adaptations such as camouflage and mimicry. The epigenesis may also explain the variation and heredity of complex traits and diseases in humans [100].

According to Lynn Margulis the most important force in organic evolution is the endosymbiosis where bacteria have played a fundamental role in the origin of mitochondria and the chloroplasts. But, the roles of ncRNAs, their relation to RNA viruses embedded in the genome of species belonging to the three domains of the tree of life show that the endosymbiotic model must be extended to viruses. The phylogenetic analysis of relationships between RNA virus sequences and their hosts reveal coespeciation, products of thousands of years of ecological interaction between them. For these reasons it is necessary that the study of genome evolution, including human evolution, must include viruses and other parasites that also evolved from the same environments [101].
